# Analysis of sagittal alignment parameters following anterior cervical hybrid decompression and fusion of multilevel cervical Spondylotic myelopathy

**DOI:** 10.1186/s12891-018-2378-y

**Published:** 2019-01-05

**Authors:** Yuming Huang, Zhibin Lan, Weihong Xu

**Affiliations:** Department of Spine Surgery, The First Affiliated Hospital of Fujian Medical University, Fuzhou, Fujian China

**Keywords:** ACHDF, Sagittal alignment, C2–7 lordosis; C2–7 SVA, T1 slope; cervical tilting

## Abstract

**Background:**

To investigate the relationships between sagittal parameters and health-related quality of life (HRQOL) scores following anterior cervical hybrid decompression and fusion (ACHDF) of multilevel cervical spondylotic myelopathy (CSM) and to study the impact of the T1 slope (T1 s).

**Methods:**

In total, 42 patients with complete radiographic measurements following ACHDF in the Spine Surgery Department of the First Affiliated Hospital of Fujian Medical University from August 2014 to January 2017 were retrospectively analysed. Radiographic measurements included C2–7 lordosis, T1 s, C2–7 sagittal vertical axis (SVA), cervical tilting and cranial tilting. The neck disability index (NDI) was used to evaluate the HRQOL. Spearman’s correlation coefficients were calculated between pairs of cervical sagittal parameters and NDI scores.

**Results:**

Preoperative NDI scores were correlated with preoperative T1 s (*r* = 0.413); follow-up NDI scores were correlated with follow-up T1 s (*r* = 0.534). The regression analysis indicated that a preoperative T1 s value of 42.36° corresponded to a preoperative NDI score of 25 (r^2^ = 0.171, *P* < 0.001). A follow-up T1 s value of 48.61° corresponded to a follow-up NDI score of 25 (r^2^ = 0.421, *P* < 0.01). The differences in C2–7 SVA and cranial tilting before and after the operation were statistically significant (*P* < 0.05).

**Conclusion:**

This study showed that the sagittal balance of the cervical vertebrae changed significantly after ACHDF, showing a forward trend. The sagittal parameters after ACHDF were related to clinical prognosis. An excessive T1 s can be considered a risk factor. The T1 s could provide a reference value to determine the correction of the sagittal balance of the cervical spine.

## Background

Anterior cervical hybrid decompression and fusion (ACHDF) can decompress the anterior cord, reduce the amount of surgical bleeding and maintain the stability of the spinal column [1, 2]. Studies have revealed that 3-level anterior cervical discectomy and fusion (ACDF) is associated with a higher incidence of nonunion due to increased graft-host interfaces [3], whereas 2-level ACDF has a higher rate of device-related complications [4, 5]. Therefore, combining anterior cervical corpectomy and fusion (ACCF) and ACDF has been introduced as an alternative procedure because it may avoid some drawbacks of traditional fusion techniques [6]. However, a force line may be altered due to surgical discectomy and corpectomy, which may affect the sagittal balance and clinical prognosis.

Until now, there has been no standard for the indication and degree of correction of the sagittal balance of the cervical spine. The T1 slope (T1 s) is an emerging prospective parameter proposed by Lee [7]. It is analogous to the sacrum slope (SS) of the lumbosacral segment and represents the degree of forward tilt of the cervical spine. Current research suggests that maintaining a small T1 s value can maintain the sagittal stability of the cervical spine [8, 9]. An increase in T1 s will increase the energy consumption of the posterior muscle ligament of the cervical spine, resulting in unbearable neck pain symptoms in some patients [10]. However, no related study has examined the proximity threshold of patients with increased T1 s. The purpose of this study was to explore the relationship between the sagittal parameters of ACHDF after multilevel cervical degeneration and health-related quality of life (HRQOL) scores and to explore the impact of the T1 s, which may provide a reference value for determining the correction of the sagittal balance of the cervical spine.

## Methods

After obtaining institutional review board approval, a retrospective analysis of clinical and radiographic outcomes was performed for patients who received ACHDF of multilevel (3 or more) cervical spondylotic myelopathy (CSM) in the Spine Surgery Department of the First Affiliated Hospital of Fujian Medical University from August 2014 to January 2017. All patients with complete radiographic measurements were diagnosed by a detailed inquiry regarding their medical history, an imaging examination and a physical examination. Subtotal corpectomy of the vertebral body was performed based on the patient’s main symptoms, major severe compression segments, and ossification of the posterior longitudinal ligament, and discectomy was performed based on secondary symptoms and compression segments. The inclusion criteria included the following: (1) cervical stenosis identified on the imaging examination; (2) no prior cervical spine procedures or insertion of instrumentation into the cervical spine; and (3) available and complete preoperative, postoperative and follow-up lateral standing cervical radiographs. Patients who had experienced trauma or who had tumours or infections of the spine were excluded; patients for whom it was difficult to measure the sagittal alignment parameters were also excluded (the T1 vertebral body was not clearly visible on the X-ray or measurement of the vertebral body was blocked by the sternum or ribs in the sagittal plane).

**Fig. 1:**
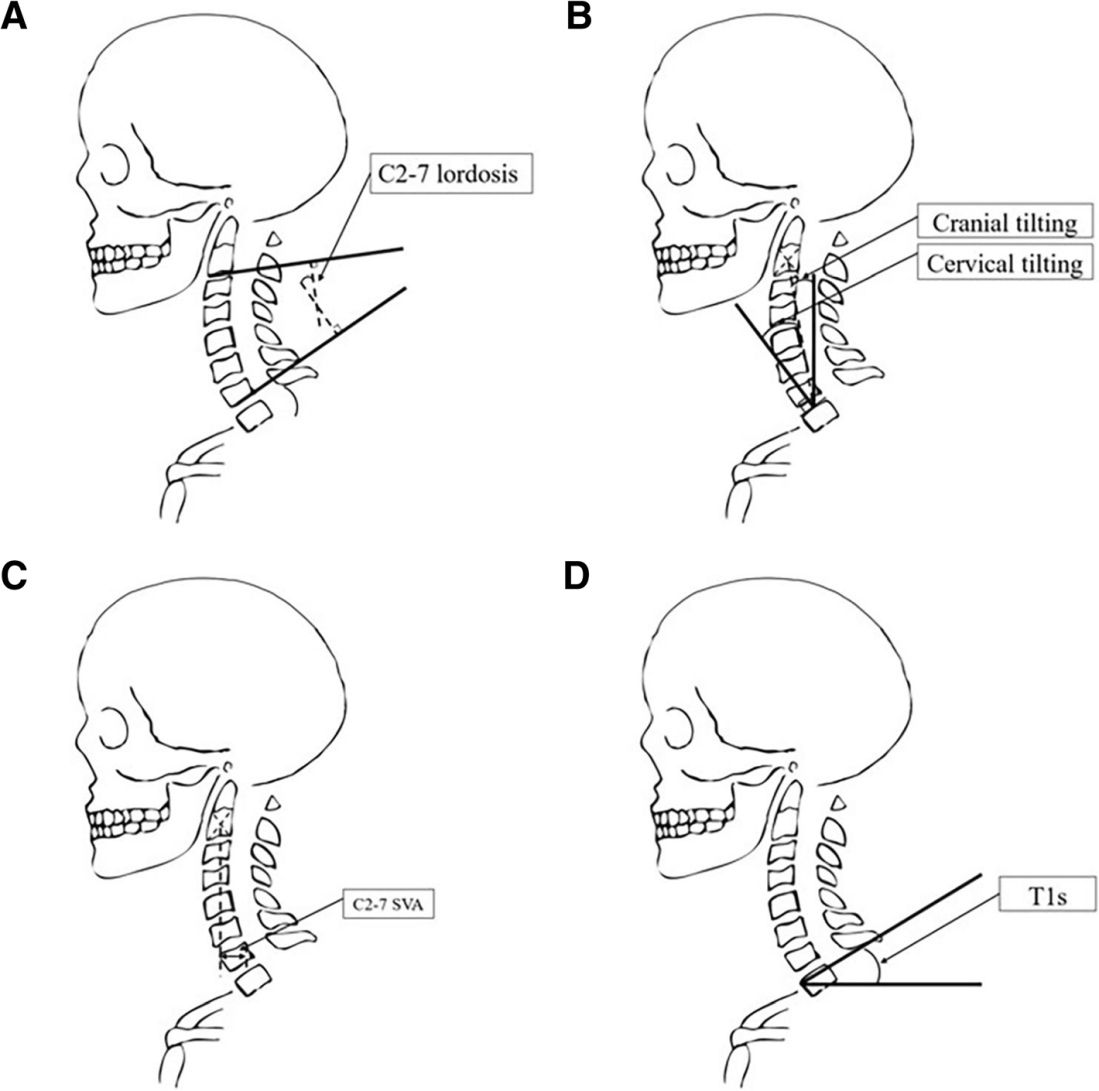
Measurements of parameters. **a** C2–7lordosis; **b**: Cranial tilting and cervical tilting; **c**: C2–7SVA, sagittal vertical axis; **d**: T1 s,T1 slope

Radiographs were taken using a standard technique and the same machine in the standing position and uploaded to the PACS system at our institution. In order to avoid the measurement error caused by incorrect posture, in the process of filming the lateral radiograph of the cervical vertebra, the imaging doctor will strictly explain the patient directly in front of the patient, and the T1 s emphasized in this study are compared with other vectors. The advantage of the face is that it does not affect the receptor site, which is also mentioned in Hey’s article [11]. The radiographic parameters examined included the following (Fig. 1): (1) C2–7 lordosis: the angle created by a line parallel to the inferior aspect of the C2 body and a line parallel to that of the C7 body; (2) C2–7 sagittal vertical axis (SVA): the distance between the plumb line dropped from the centroid of C2 and the posterior superior aspect of C7; (3) T1 s [7]: the angle between a horizontal line and the superior endplate of T1; (4) cervical tilting [7]: the angle between two lines, both originating from the centre of the T1 upper endplate; one is vertical to the T1 upper endplate and the other passes through the tip of the dens; and (5) cranial tilting [7]: the angle between two lines, both originating from the centre of the T1 upper endplate, with one passing through the dens and the other being a line perpendicular to the T1 endplate. The difference between the preoperative and postoperative values for each parameter was designated as the △ value. The self-assessment NDI scores were obtained from each patient. The neck disability index (NDI) scores were evaluated by categorizing them into the following standard intervals: no disability (0–4), mild (5–14), moderate (15–24), severe (25–34), and complete disability (> 34). The clinical prognosis was assessed by the NDI scores, which were collected in preoperation and at least 1 year after surgery.

All data were statistically analysed with SPSS 23.0 software. Measurement data are expressed as the□x ± s. All parameters exhibited a normal distribution. Spearman’s correlation coefficient and linear regression analysis were calculated for the radiographic parameters and NDI scores, and partial correlation coefficients were calculated to quantify the confounding factors for the association between the sagittal parameters and NDI scores. A paired T-test was used to compare the effects of ACHDF of multilevel CSM on the cervical sagittal alignment parameters. A value of *P* < 0.05 was considered statistically significant.

## Results

### Demographic data

In total, 42 patients (M = 30, F = 12) were identified, and the mean age was 55.9 ± 10.9 years. The involved segments were C3–6 (18 cases) and C4–7 (24 cases). The average Body Mass Index (BMI) was 24.33 ± 2.93 kg/m2 (Table 1). The average blood loss was 118.6 ± 111.7 ml, and the average operative time was 134.9 ± 62.0 min. The average postoperative follow-up period for which radiographic measurements and NDI scores were obtained was 20.3 ± 8.1 months.

**Table 1:** General Information of patients

Item	Group ACHDF
Case	42
Average age (years)	55.9 ± 10.9
Sex (male:female)	30: 12
BMI (kg/m2)	24.33 ± 2.93
Surgical segment (cases)	C3–6: 18
	C4–7: 24
Average follow-up (months)	20.3 ± 8.1

### Effects on cervical sagittal alignment parameters

The preoperative T1 s was 26.36 ± 8.16°, the postoperative T1 s was 26.94 ± 7.00°, and the follow-up T1 s was 27.69 ± 6.49°. The preoperative C2–7 SVA was 1.37 ± 1.41 cm, the postoperative C2–7 SVA was 1.73 ± 1.23 cm, and the follow-up C2–7 SVA was 1.64 ± 1.14 cm. The preoperative C2–7 lordosis was 15.13 ± 10.74°, the postoperative C2–7 lordosis was 15.81 ± 8.18°, and the follow-up C2–7 lordosis was 16.82 ± 7.13°. The preoperative cranial tilting was 5.53 ± 6.33°, the postoperative cranial tilting was 7.57 ± 7.38°, and the follow-up cranial tilting was 8.51 ± 6.63°. The preoperative cervical tilting was 18.14 ± 7.40°, the postoperative cervical tilting was 16.61 ± 7.14°, and the follow-up cervical tilting was 17.71 ± 6.36° (Table 2).

**Table 2:** The effects on cervical sagittal alignment parameters following ACHDF

Item	Pre-	Post-	Follow-up	*P* value	*P*▲ value
C2–7SVA (cm)	1.37 ± 1.41	1.73 ± 1.23	1.64 ± 1.14	0.019*	0.414
C2–7lordosis (°)	15.13 ± 10.74	15.81 ± 8.18	16.82 ± 7.13	0.614	0.265
T1 s (°)	26.36 ± 8.16	26.94 ± 7.00	27.69 ± 6.49	0.621	0.421
Cervical tilting (°)	5.53 ± 6.33	7.57 ± 7.38	8.51 ± 6.63	0.025*	0.205
Cranial tilting (°)	18.14 ± 7.40	16.61 ± 7.14	17.71 ± 6.36	0.725	0.179

(*P* value, comparison between pre- and post-; *P*▲ value, comparison between post- and follow-up; ACHDF, anterior cervical hybrid decompression and fusion; SVA, sagittal vertical axis; T1 s, T1 slope)

△T1 s exhibited a significant correlation with △C2–7 lordosis (*r* = 0.334) and △cervical tilting (*r* = 0.391). △C2–7SVA exhibited a significant correlation with △cranial tilting (*r* = 0.605). △Cervical tilting exhibited a significant correlation with △C2–7 lordosis (*r* = 0.502) and △cranial tilting (*r* = − 0.622) (Table 3).

**Table 3:** Correlation between the changes of sagittal alignment parameters following ACHDF

		△T1 s	△C2–7SVA	△C2–7 lordosis	△Cranial tilting	△Cervical tilting
△T1 s	r		0.118	0.334*	− 0.063	0.391**
	P		0.458	0.031	0.692	0.010
△C2–7SVA	r	0.118		−0.132	0.605**	− 0.218
	P	0.458		0.406	0.000	0.165
△C2–7 lordosis	r	0.334*	− 0.132		−0.310*	0.502*
	P	0.031	0.406		0.045	0.001
△Cranial tilting	r	−0.063	0.605**	− 0.310*		−0.622**
	P	0.692	0.000	0.045		0.000
△Cervical tilting	r	0.391**	− 0.218	0.502**	− 0.622**	
	P	0.010	0.165	0.001	0.000	

ACHDF, anterior cervical hybrid decompression and fusion; SVA, sagittal vertical axis; T1 s, T1 slope

*Correlation is significant at the *P* < 0.05 level (2-tailed);

**Correlation is significant at the *P* < 0.01 level (2-tailed)

### Correlations between parameters and NDI scores

**Fig. 2:**
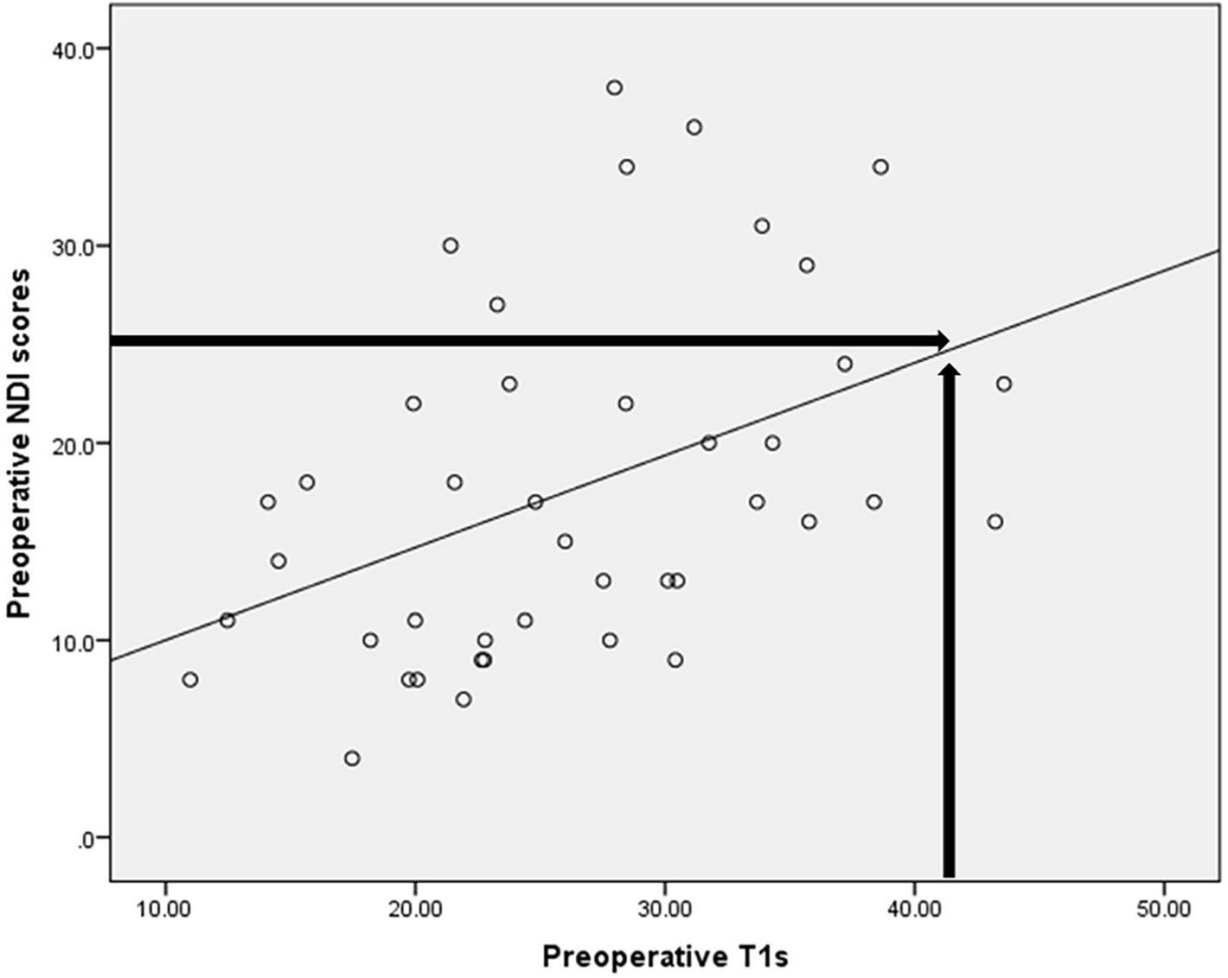
The linear regression between preoperative T1 s and NDI scores. The linear regression predicted a threshold T1 s value of 41.85° for an NDI score of 25 at preoperation

**Fig. 3:**
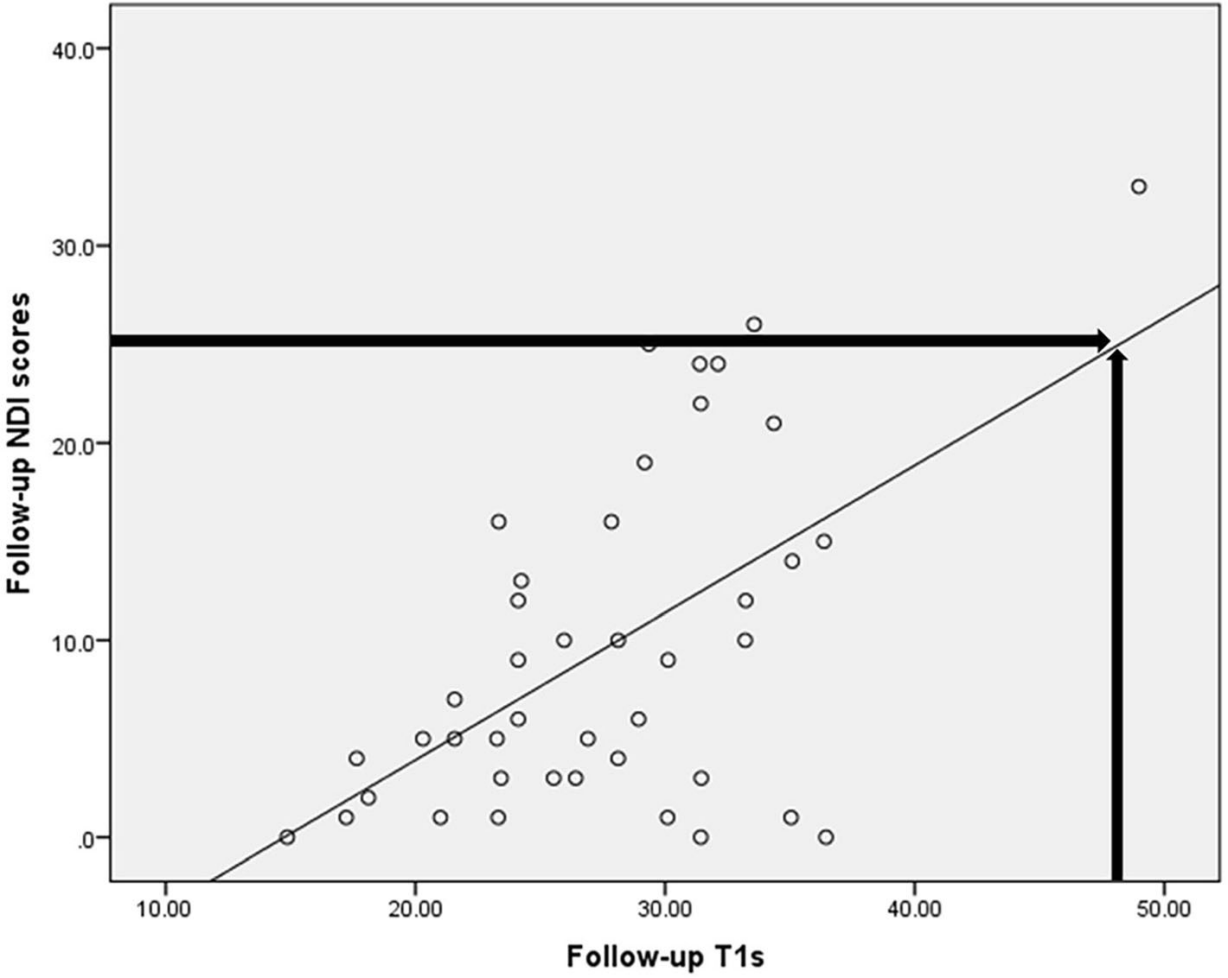
The linear regression between Follow-up T1 s and NDI scores. The linear regression predicted a threshold T1 s value of 48.02° for an NDI score of 25 at follow-up

Preoperatively, the average NDI was 17.7 ± 8.8 (4–38). NDI scores were significantly correlated with the T1 s (*r* = 0.489, *P* < 0.01); the linear regression predicted a threshold T1 s value of 41.85° for an NDI score of 25 (r^2^ = 0.188, *P* < 0.01). At follow-up, the average NDI score was 9.7 ± 8.6 (0–33). The follow-up NDI score was significantly correlated with the follow-up T1 s (*r* = 0.421, *P* < 0.01) (Fig. 2); the linear regression predicted a threshold T1 s value of 48.02° for an NDI score of 25 (r^2^ = 0.315, *P* < 0.001) (Fig. 3, Table 4).

**Table 4:** Correlation between sagittal parameters and NDI scores

	Sagittal parameters	Correlation (Spearman r)	*P*
Preoperative NDI scores	Preoperative T1 s	0.489	0.001**
	Preoperative C2–7SVA	0.206	0.191
	Preoperative C2–7 lordosis	0.245	0.117
	Preoperative Cranial tilting	0.154	0.331
	Preoperative Cervical tilting	0.449	0.003**
Follow-up NDI scores	Follow-up T1 s	0.421	0.006**
	Follow-up C2–7SVA	0.106	0.503
	Follow-up C2–7 lordosis	0.190	0.228
	Follow-up Cranial tilting	0.115	0.470
	Follow-up Cervical tilting	0.156	0.325

*SVA* sagittal vertical axis, *T1 s* T1 slope

**Correlation is significant at the *P* < 0.01 level (2-tailed)

The partial correlation coefficient was used to control this confounding variable. According to the partial correlation analysis, the preoperative T1 s was positively correlated with the preoperative NDI scores (*r* = 0.361, *P* < 0.05), and the follow-up T1 s was also positively correlated with the follow-up NDI scores (*r* = 0.515, *P* < 0.01). However, there was no correlation between age and follow-up NDI scores (Table 5).

**Table 5:** Partial correlation coefficient

Item	Item	No adjust		Adjust-age/T1 s	
		r	*P*△value	r	*P*▲value
Preoperative NDI	Preoperative T1 s	0.489	0.001**	0.361	0.020*
Follow-up NDI	Follow-up T1 s	0.421	0.006**	0.515	0.001**
Preoperative NDI	Age	0.440	0.004**	0.316	0.044*
Follow-up NDI	Age	0.332	0.032*	0.176	0.270
Preoperative T1 s	Age	0.343	0.026*		
Follow-up T1 s	Age	0.340	0.027*		

*P△value* spearman correlation between two items, *P▲value* partial correlation between two items, *T1 s* T1 slope

*Correlation is significant at the *P* < 0.05 level (2-tailed);

**Correlation is significant at the *P* < 0.01 level (2-tailed)

## Discussion

In recent years, the influence of important parameters on HRQOL scores has attracted increasing attention from scholars [12–14]. In this study, we aimed to confirm the relationship between cervical sagittal parameters and NDI scores after ACHDF of multilevel CSM and to explore the impact of T1 s, which may provide a reference value for determining the correction of the sagittal balance of the cervical spine.

It is well known that C2–7 SVA is an important parameter for predicting cervical surgery outcomes. Tang et al. [13] confirmed that C2–7 SVA was significantly correlated with NDI scores (*r* = 0.20, *P* = 0.036) in multilevel posterior cervical fusion, and regression models predicted a threshold C2–7 SVA value of 40 mm. Smith et al. [15] found that C2–7 SVA was negatively correlated with JOA scores for cervical spondylotic myelopathy. The T1 s is known to have a significant correlation with the C2–7 SVA [16].

C2–7 lordosis is also a common index used to assess cervical curvature. Many scholars have considered that maintaining a lordosis after surgery results in a positive outcome, and the possible reason for this result is that the neck muscles and ligaments surrounding the cervical spine may maintain a lower energy expenditure [17–21]. In our study, △C2–7 lordosis was also correlated with △T1 s (*r* = 0.334).

The T1 s was affected by not only the lower cervical spine but also the upper cervical spine. Lee et al. [7] also found that the ratio of cervical tilting to cranial tilting was 70.2%: 29.8% in asymptomatic subjects, which indicates that when the two-party ratio was kept at 7:3, the energy consumption could be reduced to a minimum. Therefore, T1 s is a key parameter for cervical sagittal balance.

Furthermore, In this study, the T1 s was also significantly positively correlated with NDI scores both preoperatively and postoperatively (*r* = 0.489 and *r* = 0.421, respectively). In particular, we demonstrated that a preoperative T1 s greater than 42° or a follow-up T1 s greater than 48° indicated a poor clinical prognosis, defined as an NDI score greater than 25. Moreover, it supports the cervical spine anatomically and biomechanically through the attachment of various muscles around the neck and is therefore a key parameter affecting the NDI scores.

Although most surgeons will not ignore the risk factor of age, the age-related change in cervical sagittal parameters may not be considered. Therefore, a partial correlation coefficient was used to control the confounding variable of age; the conclusion was that T1 s was still significantly correlated with the preoperative NDI scores and follow-up NDI scores. Thus, the T1 s may be a very important parameter for determining the clinical prognosis.

In recent surveys, Shin Oe et al. [8] found that patients with a T1 s > 40° had poorer outcomes than those with smaller T1 s values because patients with larger T1 s values may have decompensated changes in the cervicothoracic spine. In patients with cervical laminoplasty, Kim et al. [14] found that high a T1 s was associated with a positive sagittal imbalance. These results agree with our current research. A biomechanical study conducted by Patwardhan et al. [10] found that patients with forward head posture (FHP, high T1 s and C2–7SVA) required more physical work from the suboccipital muscles and that increasing the T1 s had the predominant effect of increasing C2–7 lordosis, which meant that more posterior neck muscles were shortened. Finally, contracted states may result in painful trigger points, leading to some of the neck pain associated with a large T1 s.

According to our research, the operation directly changed the C2–7 SVA parameter and cervical tilting, indicating that patients tended to tilt the cervical spine forward after a routine ACHDF operation. Furthermore, △T1 s was significantly correlated with △C2–7 lordosis (*r* = 0.334) and △cervical tilting (*r* = 0.391); thus, the reduction of the angle of cervical tilting or C2–7 lordosis in the operation could indirectly reduce the T1 s. It is not difficult to understand this correlation; as a key vertebra, T1 joins the cervical and thoracic vertebra, and it is fixed on both sides of the ribs and not altered by position. Therefore, the T1 s parameter may provide a reference value for determining the correction of the sagittal balance of the cervical spine. But it is more complicated in the clinical setting. The surgical strategy to improve the clinical prognosis cannot simply discuss the preoperative and postoperative parameters of the sagittal plane but must also consider the patient’s clinical symptoms and the positional compression.

**Fig. 4:**
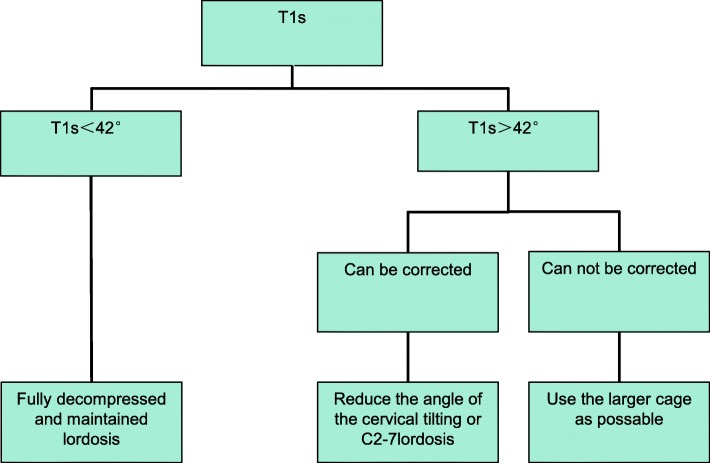
Surgical decision-making flow chart for patients with cervical spondylosis. T1S, T1 slope

Therefore, we classified multilevel CSM patients into different groups (Fig. 4) as follows: 1. T1 s < 42°, fully decompressed and maintained lordosis can effectively improve the clinical symptoms of patients; 2. T1 s > 42°, that cannot be corrected easily (obese, neck is too short or poor activity level); perhaps we could choose the larger cage as much as possible in the operation (the cage models commonly used in cervical surgery are 5 mm, 6 mm and 7 mm), and increase the C2–7 lordosis, which may bring better clinical prognosis for patients, which seems to increase the negative impact of T1 s, but according to Kim [14] and Hyun et al. [12], the T1 s-CL (T1 s minus C2–7 lordosis) is also reduced. We completely agree with their point of view. Patients with lower T1 s-CL could also have significantly improved functional recovery. When the patient maintains a small T1 s-CL, the suboccipital muscle group and the neck muscle group are mostly in the relaxed state, which significantly improves postoperative axial pain; when the T1 s-CL decreases, the patient’s neck is tilted backward, which restores the visual field and maintains the balance of the cervical spine. and 3. T1 s > 42°, that could be corrected easily during operation by the X-ray; suitable cages are need to decrease the T1 s by decreasing the cervical tilt or C2–7 lordosis intraoperatively. It is well known that the T1 s of most patients does not exceed the threshold. In this regard, we believe that in a routine ACHDF operation, it is more important to improve the patient’s clinical prognosis by keeping a slightly forward position.

The limitations of this study include a small sample size. Due to the retrospective study design, this study used the NDI score because the clinical prognosis and quality of life index were too individual, and the JOA score and SF-36 were not evaluated. In addition, the sagittal position of the cervical spine is associated with many types of demographic data, and several confounding factors may affect the outcome, we also lacked of a control group such as 3-level ACDF patients. It is therefore hoped that follow-up studies can enhance and verify the conclusions of this article by increasing the sample size and having a longer follow-up period. Follow-up studies could also use a prospective double-blind study design and additional scoring criteria to address the impact of the sagittal parameters on the prognosis of patients.

## Conclusions

This study showed that the sagittal balance of the cervical vertebrae changed significantly after ACHDF, showing a forward trend. The sagittal parameters after ACHDF were related to the clinical prognosis. An excessive T1 s can be considered a risk factor. The T1 s could provide a reference value to determine the correction of the sagittal balance of the cervical spine.

## Abbreviations

ACCF: Anterior cervical corpectomy and fusion
ACDF: Anterior cervical discectomy and fusion
ACHDF: Anterior cervical hybrid decompression and fusion
BMI: Body Mass Index
CSM: Cervical spondylotic myelopathy
HRQOL: Health-related quality of life
NDI: Neck disability index
SVA: Sagittal vertical axis
T1 s: T1 slope
